# Quality of Life and Psychological Well-being in Children and Adolescents with Disorders of Sex Development

**DOI:** 10.4274/jcrpe.galenos.2020.2020.0141

**Published:** 2021-02-26

**Authors:** Birsen Şentürk Pilan, Burcu Özbaran, Didem Çelik, Tuğçe Özcan, Samim Özen, Damla Gökşen, İbrahim Ulman, Ali Avanoğlu, Sibel Tiryaki, Hüseyin Onay, Özgür Çoğulu, Ferda Özkınay, Şükran Darcan

**Affiliations:** 1 Ege University Faculty of Medicine, Department of Child and Adolescent Psychiatry, İzmir, Turkey; 2 Ege University Faculty of Medicine, Department of Pediatric Endocrinology, İzmir, Turkey; 3 Ege University Faculty of Medicine, Department of Pediatric Surgery, İzmir, Turkey; 4 Ege University Faculty of Medicine, Department of Medical Genetics, İzmir, Turkey

**Keywords:** Disorder of sex development, quality of life, psychiatric disorder, child and adolescent

## Abstract

**Objective:**

The aim of this study was to assess the quality of life (QoL) and psychological well-being in child and adolescent with disorders of sex development (DSD).

**Methods:**

Sixty-two cases, aged 2-18 years, who were followed by a multidisciplinary DSD team were included. All participants and their parents were requested the complete the Pediatric Quality Of Life Inventory (PedsQL) and the Strengths and Difficulties Questionnaire. The psychiatric diagnoses of the patients were evaluated according to Schedule for Affective Disorders and Schizophrenia for School-Age Children/Present and Lifetime Turkish Version.

**Results:**

There was no significant difference between the 46,XX DSD and 46,XY DSD groups for both child and parent in Total PedsQL score. In the subscale scores, the PedsQL Physical Functionality Score reported by children was significantly lower for the 46,XX DSD group than for the 46,XY DSD group (p=0.01). There was a psychiatric diagnosis in 25.8% of cases. The PedsQL School Functionality Score reported by children in the group with psychiatric diagnosis was significantly lower than the group without psychiatric diagnosis (p=0.018). In the group with psychiatric diagnosis, the PedsQL Total Score and the subscale scores (Emotional Functionality Score, Social Functionality Score, School Functionality) reported by parents were significantly lower than in parents of the group without psychiatric diagnosis.

**Conclusion:**

This study emphasized that psychiatric disorders in DSD patients negatively affect the QoL. Psychiatric support and counseling from a multidisciplinary team are very important for families affected by DSD.

**What is already known on this topic?**

Articles about quality of life (QoL) in disorders of sex development (DSD) patients who were evaluated with qualitative and quantitative tools in developing and developed countries were reviewed. A broad spectrum of QoL emerged with results better, worse or similar for QoL compared with the unaffected population. No study from Turkey evaluating the QoL of children and adolescents with DSD was found.

**What this study adds?**

In our study, there was no significant difference between 46,XX DSD and 46,XY DSD groups for both child and parent using the total Pediatric Quality of Life Inventory (PedsQL) scores. In the subscale scores, the PedsQL Physical Functionality Score of affected children was significantly lower in the 46,XX DSD group than in the 46,XY DSD group (p=0.01). PedsQL School Functionality Score, reported by children and adolescents, was significantly lower in the group with a psychiatric diagnosis compared to the group without a psychiatric diagnosis. In addition, the PedsQL Total Score, PedsQL Emotional Functionality Score, PedsQL Social Functionality Score, and PedsQL School Functionality Score reported by parents in the group with psychiatric diagnosis were significantly lower than the group without psychiatric diagnosis. These results suggest that psychiatric disorders in patients with DSD are the most important factor affecting the QoL.

## Introduction

Rare congenital conditions that are characterized by incompatibility of chromosomal, gonadal, and phenotypic gender characteristics are classified as disorders of sex development (DSD) (1). The incidence of DSDs is approximately 1 in 4500-5500 (2). Studies to date have focused on psychosexual outcomes, such as gender dysphoria, sexual function status and satisfaction of surgical outcomes in individuals with DSD but very few have assessed general well-being or social participation (3). Long-term psychological, physical and social consequences of young people with DSD are uncertain (4).

Health-related quality of life (HRQOL) has a multidimensional structure that includes various core states, including physical functionality and symptoms, psychological and emotional state and social functionality, that reflect subjective assessments of the patient and his/her family (5). HRQOL measures are increasingly used to determine the impact of medical interventions on compliance and psychosocial well-being (6,7). Important information to guide sex assignment in newborns with indeterminate genital organs is the QoL of these patients in adulthood. The rareness of occurrence of most DSD conditions complicates long-term follow-up of affected patients during adulthood. In the study of Amaral et al (8), articles concerning the QoL in DSD patients who were evaluated with both qualitative and/or quantitative tools in developing and developed countries were reviewed. A broad spectrum of QoL emerged with results better, worse or similar for QoL compared with the unaffected population.

In addition, most of the patients’ dissatisfaction was not associated with poor management of the disease or with the assigned gender. A better understanding of their condition, and co-operation between the family and medical team lead to increased satisfaction with treatment. The review of Amaral et al (8) showed that a talented, multidisciplinary team is necessary to deal with these patients throughout their diagnosis and life, and co-operation with patients and parents is crucial.

There is no study from our country evaluating the QoL of children and adolescents with DSD, to the best of our knowledge. In our study, it was aimed to evaluate the HRQOL in children and adolescents with DSD and their parents, in order to better understand future health interventions and approaches.

## Methods

In this one-year study 62 cases aged 2-18 years who were followed by multidisciplinary DSD team and were referred to Ege University Department of Child and Adolescent Mental Health and Diseases were included. All participants and their parents were requested to complete the Pediatric Quality of Life Inventory (PedsQL). The Strengths and Difficulties Questionnaire (SDQ), related to emotional and behavioral problems, was completed by parents and teachers of 4-17 year-old cases and, in addition, in patients aged above 11 years old the SDQ was also self-completed. The psychiatric diagnoses of the patients were evaluated according to Schedule for Affective Disorders and Schizophrenia for School-Age Children/Present and Lifetime Turkish Version (K-SADS-PL-T) and Diagnostic and Statistical Manual of Mental Disorders-5 (DSM-5) diagnostic criteria.

The classification of the medical diagnosis of patients with DSD was made according to the Lawson Wilkins Pediatric Endocrine Society and the European Society for Pediatric Endocrinology Consensus Statement (9).

In order to make statistical comparisons between groups, endocrine diagnoses were grouped into four groups: 46,XX DSD Group, 46,XY DSD Group, Syndromic Group and Chromosomal Disorder Group. However, since there were not enough cases in the Syndromic and Chromosomal Disorder groups they were removed during the statistical evaluation and the comparisons between the scales were made between the 46,XY DSD and 46,XX DSD groups.

Socio-demographic data, including age, gender, school, mother’s and father’s education were recorded in the case data form prepared by the authors. Following a full description of the study and study procedure, patients who could give informed consent and all parents were asked to provide written consent. The study was approved by Ege University Medical Research Ethics Committee (19-10.1T/56, 16.10.2019).

### Tools

**K-SADS-PL-T:** A semi-structured interview form, K-SADS-PL-T was developed by Kaufman et al (10) (1997) in order to determine the past and present psychopathologies of children and adolescents according to DSM-5 (11) diagnostic criteria. The validity and reliability study for the Turkish language version was conducted by Gökler et al (12) (2004). In K-SADS-PL-T, the presence and severity of symptoms are determined by combining the views of the child/adolescent, parents and clinician. During the study period two clinicians confirmed the psychiatric diagnoses according to DSM-5 diagnostic criteria (13).

**PedsQL:** HRQOL was assessed using the PedsQL which contains 23 items in four subscales, including physical (eight items), emotional (five items), social (five items) and school (five items) functioning. There are four different forms of the scale for the 2-4, 5-7, 8-12 and 13-18 age groups. Children rated how often the item has been a problem for them in the past one month using a five-point response-scale format (0=never a problem, 1=almost never a problem, 2=sometimes a problem, 3=often a problem, 4=almost always a problem). The scores ranged from 0 to 100, with higher scores indicating better HRQOL. The total PedsQL score was computed as the sum of all items divided by the number of items answered. For the PedsQL internal consistency (Cronbach alfa=0.70-0.89) and clinical reliability are high (14). The reliability and validity of the Turkish version of PedsQL in adolescents (for the 8-12 and 13-18 age groups) were reported by Cakin Memik et al (15) and Memik et al (16) while the versions for 2-7 year olds was validated by Üneri. (17)

**SDQ:** This scale is used in screening emotional and behavioral problems in children. It was developed by Goodman (18) in 1997 and it contains 25 questions. These questions are under five subtitles, each of five questions; Emotional Problems, Attention Deficit and Hyperactivity, Behavioral Problems, Peer Problems and Social Behaviors. This questionnaire has a parent and teacher form for ages 4-17 and an adolescent’s self-filled forms for ages 11-17. The Turkish validity and reliability study of this questionnaire for both the parent and adolescent forms was conducted (19,20). However, the Turkish validity and reliability has never been confirmed, to the best of our knowledge.

### Statistical Analysis

Statistical analysis was done using SPSS, version 22 (IBM Inc., Armonk, NY, USA. The normality assumption of quantitative data was assessed in each group by Shapiro-Wilk test. The statistical significance was investigated using t-test for numerical variables, Mann-Whitney U test for non-normal distributions, cross table, Pearson chi-square test and Fisher’s exact test for categorical variables. A p<0.05 was considered statistically significant. Variable correlation was evaluated by Pearson correlation, if normal distribution was detected, and Spearman correlation if non-parametric.

## Results

The average age of 62 cases participating in the study was 9.70±4.18 years. Thirty-six (58.1%) of the cases were raised in the female sex and 26 (41.9%) in the male sex. Socio-demographic characteristics are summarized in Table 1.

Endocrine diagnoses of the cases were: 46,XX DSD in 30.6% (n=19), 46,XY DSD in 67.7% (n=42) and chromosome disorders in 1.6% (n=1). Endocrine diagnoses are summarized in Table 2.

There was a psychiatric diagnosis in 16 (25.8%) cases, and there was no psychiatric diagnosis in the remaining 46 (74.2%). The mean age of patients with a psychiatric diagnosis was 11±4.02 years, and the mean age of those without a psychiatric diagnosis was 9.26±4.17 years. The most common psychiatric diagnosis was attention deficit and hyperactivity disorder (ADHD) (=13, 21.0%). The other psychiatric diagnoses were depressive disorder (n=2, 3.2%), mental retardation (n=2, 3.2%), anxiety disorder (n=3, 4.8%), autism (n=1, 1.6%), and specific learning disability (n=1, 1.6%).

**PedsQL and SDQ Scores:** Scale score comparisons were made between 46,XY DSD and 46,XX DSD groups.

Patient diagnoses in the 46,XX DSD group included CAH (n=15, 24.2%), virilizing tumor lutheoma in mother (n=1, 1.6%), 46,XX DSD with no diagnosis (n=2, 3.2%) and ovotesticular 46,XX DSD (n=1, 1.6%) diagnoses were included.

Diagnoses in the 46,XY DSD group were partial gonadal dysgenesis (n=5, 8.1%), complete gonadal dysgenesis (n=5, 8.1%), androgen synthesis defects (n=17, 27.4%), partial androgen insensitivity syndrome (n=2, 3.2%), complete androgen insensitivity syndrome (n=3, 4.8%), persistent Müllerian duct syndrome (n=3, 4.8%) and 46,XY with no diagnosis (n=3, 4.8%) were included.

There was no significant difference between 46,XX DSD and 46,XY DSD groups in both child and parent total PedsQL scores. In the subscale scores, the PedsQL Physical Functionality Score (PFS) reported by children was significantly lower in the 46,XX DSD group than in the 46,XY DSD group (p=0.01).

When the relationship between the sex in which the child was raised and the QoL scores was evaluated, no significant difference was found in both child and parent scores (p>0.05).

There was mental illness in the family in 8.1% (n=5) of the cases. There was no significant relationship between mental illness in the family and QoL scores of the children and parents (p>0.05).

Forty-nine (79.0%) patients had surgical intervention and 13 (21.0%) had no surgical intervention. Surgical interventions included examination, corrective operation and gonadectomy.

The QoL PFS reported by children was significantly higher in patients with surgical intervention than those without (p=0.039).

In our study 72.6% (n=45) of the cases were prepubertal and 27.4% (n=17) were pubertal. No case was sexually active. When looking at the QoL scores of the cases according to their pubertal status, no significant difference was found between prepubertal and pubertal cases in terms of QoL scores in the scales completed by children. PedsQL Emotional Functionality Score (p=0.029) and PedsQL School Functionality scores (p=0.003), among the QoL subscale scores completed by the parents, were found to be significantly lower in pubertal cases than in prepubertal cases.

Table 3 and Table 4 show the relationship between children’s and parent’s PedsQL scores and socio-demographic characteristics, endocrine groups, pubertal status and surgical intervention.

No significant correlation was found between endocrine diagnosis age (3.23±4.30 years), disease duration calculated from age of diagnosis (6.79±4.19 years) and PedsQL scores (p>0.05, Spearman correlation test).

When the relationship between patient age and QoL was examined, no significant correlation was found between age and QoL scores completed by the children. In the parental scores, PedsQL total score (p=0.012) and PedsQL school functionality (r=0.657, p<0.05) scores decreased with increasing age and there was a significant negative correlation between them (Spearman correlation test).

Hormone replacement therapy was used in 29% (n=18) of the cases. No significant difference was found between those using hormone replacement therapy and those not using hormone replacement therapy in both child and parent QoL scores (p>0.05, Mann-Whitney U Test).

Considering the QoL scores between the two most common endocrine diagnostic groups, CAH and ASD, the PedsQL PFS filled by children was significantly lower in patients with CAH than those with ASD (p=0.017).

When the SDQ scores were analyzed, the SDQ behavioral total difficulty score reported by children was found to be significantly higher in the 46,XY DSD group than the 46,XX DSD group (p=0.002).

SDQ Behavioral Total Score reported by the patients was found to be significantly lower in CAH than ASD (p=0.027). In SDQ, filled by parents, Behavioral Total Score was found to be significantly higher in CAH than in ASD (p=0.010). According to the SDQ scale filled by teachers, Emotional Total Score was found to be significantly higher in CAH than ASD (p=0.042).

### The Relationship Between Psychiatric Diagnosis, PedsQL and SDQ Scores

PedsQL School Functionality Score reported by children and adolescents in the group with psychiatric diagnosis was significantly lower than the group without psychiatric diagnosis (p=0.018). Parents of the group with psychiatric diagnosis reported the PedsQL Total Score, PedsQL Emotional Functionality Score, PedsQL Social Functionality Score, Peds QL School Functionality to be significantly lower than parents of the group that did not have a psychiatric diagnosis (p=0.002, p=0.005, p=0.001, and p=0.001, respectively).

In the group with psychiatric diagnosis, SDQ total difficulty score filled by teachers and parents was significantly higher than the group without psychiatric diagnosis (teacher p=0.001, parent p=0.029). Table 5 shows the relationship between psychiatric diagnosis, PedsQL and SDQ scores.

Psychiatric disorders were diagnosed in 22.2% (n=10) of prepubertal cases and in 35.3% (n=6) of pubertal cases. There was no significant difference between pubertal status and presence of psychiatric diagnosis (p>0.05, chi-square test).

## Discussion

Most studies on DSD focus on children’s sex development and psychosocial well-being, sexual orientation and adult life. In the literature, the results of QoL in children and adolescents with DSD were different (21). In one study, psychological well-being and QoL were not impaired in prepubertal children with DSD (22). In our study, no significant difference was found in terms of QoL scores between prepubertal and pubertal cases in the scales completed by children. However, PedsQL Emotional Functionality Score and PedsQL School Functionality scores, which are the scores of QoL subscale completed by the parents, were found to be significantly lower in pubertal cases compared to prepubertal cases. In another study, it was reported that there is an increased risk for emotional problems in children and adolescents with DSD (23). In the results of studies with adults with DSD, although some studies have reported psychological, functional and sexual disorders (24,25,26), others have not confirmed severe restrictions or psychological problems (27,28).

Psychosexual outcomes and QoL in DSD have been most extensively studied in CAH, which accounts for about half of DSD cases. According to Kuhnle et al (29), long-term effects on general HRQOL are not expected in CAH. Johannsen et al (25), have identified lower QoL and more psychiatric symptoms in adult Danish women with CAH. Nordenskjöld et al (24) reported that both mutation type and surgical procedure affected long-term QoL for women with CAH. In a study involving both male and female CAH patients, both reported much lower scores in QoL than in the general population. The authors concluded that in both groups, poor hormone replacement therapy, obesity and sexual dysfunction may be responsible for impaired QoL (30). Children with 46,XY DSD are less extensively studied. Physical well-being is reported in most cases to not be different from the general population. However, condition-specific effects on gender identity or self-perception have been described in adolescents (21).

Most of the DSD QoL studies in the literature focused on CAH and female raised and adult cases (24,25). As far as we know, our study is the first study evaluating QoL in Turkey by comparing both 46,XX and 46,XY DSD. In their study conducted in Finland, Jaaskelainen and Voutilainen (31) found that QoL scores in cases with CAH (16 women and 16 men) were better than the control group.

In their cohort studies between female social gender and male social gender DSD patients in Brazil, Amaral et al (8), found that the adult QoL in DSD patients was good in both genders. However, they found that male social gender DSD patients with either 46,XX or 46,XY had better scores in the psychological domain than female social gender DSD patients.

When looking at QoL studies in children and adolescents with DSD, in their studies on 60 adolescents aged 13-16, Kleinemeier et al (21) found that general psychological well-being was not affected. In their study, Jürgensen (22) reported that children aged 8 to 12 years with DSD had significantly lower scores in self-completed HRQOL than those without DSD and notable deficits were reported in self-esteem, physical health and school functionality dimensions. Comparison of HRQOL between the diagnostic endocrine groups revealed no significant group differences.

In our study PedsQL PFS filled by children was found to be significantly lower in patients with CAH than those with ASD. Considering that all cases with CAH have XX karyotype and all cases with ASD have XY karyotype, this finding may be attributed to gender difference. In addition, it was observed that the QoL Physical Functioning subscale scores completed by the children were significantly lower in the 46,XX DSD group compared to the 46,XY DSD group. When these results are taken together, it suggests that QoL of cases with 46,XX, DSD in our country who were raised in the female gender was lower. This finding is different from the findings of studies conducted in other countries (8,31,21,22), in which both genders and endocrine groups were compared. It is important in terms of being the first data specific to Turkey.

In research conducted in The Netherlands, the scores of QoL reported by the parents were not impaired in any dimension (32). Similarly, in our study, no significant difference was found between the two endocrine groups in the parental total and subscale PedsQL scores. The fact that information about the QoL was obtained from both cases and parents is a strength of our study.

In our study, when the relationship between patient age and QoL was examined, no significant correlation was found between age and QoL scores completed by the children. However in the parental scores, it was found that PedsQL total score and PedsQL school functionality scores decreased with increasing age and there was a significant negative correlation between them. In the research of Jürgensen (22), the differences between the scores of children and parents, especially in terms of self-esteem, psychological and physical well-being, have been previously described for other chronic diseases (33,34). This study and the other similar studies emphasize the need for self-reporting in volunteer children who can report on themselves.

Jürgensen (22) reported that variables, such as gender identity/gender dysphoria, gender role behavior, genital surgery status of the child, number and timing of surgery or diagnosis subgroups, and knowledge of the child about the current diagnosis were not associated with decreased QoL. In our study, it was found that variables such as endocrine diagnosis age, disease duration, education status of the mother, did not affect the QoL. The PedsQL PFS reported by parents increased significantly as the education level of the father increased. It is thought that this situation may be related with the increase in understanding and coping skills as the education level increases.

In the study of Crawford et al (35), lower HRQOL was reported in patients with DSD who underwent surgery. In the research of Jürgensen (22) no relation was found between genital surgery and HRQOL. However, in our study, the PedsQL PFS filled by children was significantly higher in those with surgical intervention than those without.

Ege University Faculty of Medicine DSD multidisciplinary team consists of pediatric endocrinology, pediatric surgery, genetics, and child and adolescent psychiatry specialists. The team meets every month and discusses the patients followed up with the diagnosis of DSD and organizes follow-up and treatment. Multidisciplinary team meetings are held to ensure that the intervention in DSD cases is performed at the most appropriate time and condition. It was thought that the higher PedsQL PFS in our patients who underwent surgery might be related to this. In the studies of Migeon et al (28) thirty-nine, 46,XY DSD case were evaluated for long-term medical and surgical results using questionnaire and semi-structured interview. They concluded that most of the participants were satisfied with their body image and that there was no difference in satisfaction with their sexual functions among men and women. The authors concluded that the assignment to either sex would lead to a successful long-term outcome in most 46,XY individuals with severe genital uncertainty.

In our study, a psychiatric diagnosis was assigned to 16 (25.8%) cases. Children and adolescents with DSD are at risk, due to the difficult processes they have experienced from birth, so psychiatric evaluation is required. According to a research by Özbaran et al (36), ADHD, depression and anxiety disorder were found in DSD as psychiatric diagnoses. In the study of Jürgensen (22) it was reported that the mental health of adolescents with DSD was not affected, compared to adolescents in the control group. In previous studies of CAH patients, it has been reported that anxiety disorder and ADHD are frequently seen in these cases (37). Studies on stress and QoL levels of CAH patients show that these patients are under emotional stress that can cause depression and anxiety disorders (25). In our study the most common psychiatric diagnosis was ADHD (n=13, 21.0%). This rate is higher than the prevalence of ADHD (3.4%) reported by Polanczyk et al (38) in a meta-analysis (38). In a study assessing the prevalence of childhood psychopathology in Turkey, mental disorder prevalence was 17.1% and ADHD prevalence was 12.4% (39). The values in our study were higher for both disorders (respectively, 25.8%, 21%).

The incidence of psychiatric disorders varies according to the age group and psychiatric diagnosis. While the rates of depression and some anxiety disorders (social phobia, panic disorder) increase with adolescence, the rates of some disorders such as ADHD and separation anxiety disorder decrease (40). In our study, the mean age of patients with psychiatric disorders was 11 (±4.02) years, and the most common diagnosis was ADHD and depressive disorder.

In the research of Şan et al (41) investigating a Turkish cohort, physical health total score, psychosocial health total score and scale total score filled by parents and children were found to be statistically significantly lower in the ADHD group compared to the control group. In other studies, it has been reported that the areas of psychosocial, academic and family functionality are the most affected in children diagnosed with ADHD (42).

In our study the PedsQL School Functionality Score, which was completed by the children and adolescents was significantly lower in the group with a psychiatric diagnosis, the most common being ADHD, compared to the group without a psychiatric diagnosis. In addition, PedsQL Total Score, PedsQL Emotional Functionality Score, PedsQL Social Functionality Score, and PedsQL School Functionality Score filled by parents in the group with psychiatric diagnosis were significantly lower than the group without psychiatric diagnosis.

In the research of Sawyer et al (43), it was reported that children with psychiatric disorder had much worse HRQOL than children without psychiatric disorder in many areas and also a worse HRQL than children with physical disorders.

### Study Limitations

One of the strengths of our study is that we evaluate psychiatric disorders through a semi-structured interview. The fact that information about the QoL was obtained from both cases and parents is another strength of our study. Our study is important to emphasize that psychiatric diseases in DSD patients negatively affect the QoL. The biggest limitation of our study is that the sample size is relatively small and there is no control group. Our other limitation is that the teacher form of the SDQ questionnaire has no Turkish validity and reliability. A further limitation was that clinical severity of psychiatric illness and the presence of side effects related to drugs were not evaluated.

## Conclusion

These results suggest that psychiatric disorders in patients with DSD are the most important factor affecting QoL. To the best of our knowledge there is no other study reporting the effect of psychiatric disorder on QoL in DSD patients in Turkey. Therefore, our study is important to highlight that psychiatric illnesses in DSD patients negatively affect the QoL. Consequently, the importance of psychiatric support and consultancy of a multidisciplinary team is very clear, especially for children and families. Further studies will be important to demonstrate whether multidisciplinary team collaboration and psychiatric support have a positive effect on HRQOL in individuals with DSD.

Our results also suggest that, in Turkey, the QoL is lower in patients with 46,XX DSD who were raised in the female gender. This finding is different from the findings of studies conducted in other countries in which both genders were compared. As in many countries, it is a fact that women in our country are exposed to more risk factors than men, starting from intrauterine life, during childhood, adolescence, adulthood and old age. Future studies with larger samples and control groups will shed light on this issue.

## Figures and Tables

**Table 1:**
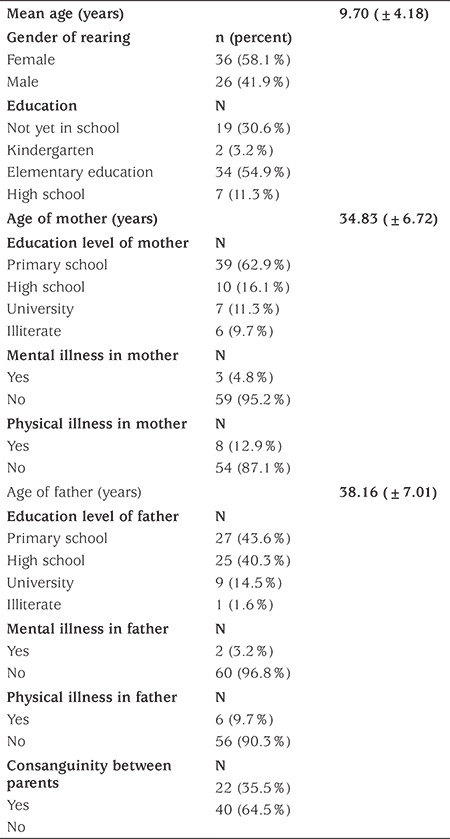
Sociodemographic characteristics of patients and their parents

**Table 2:**
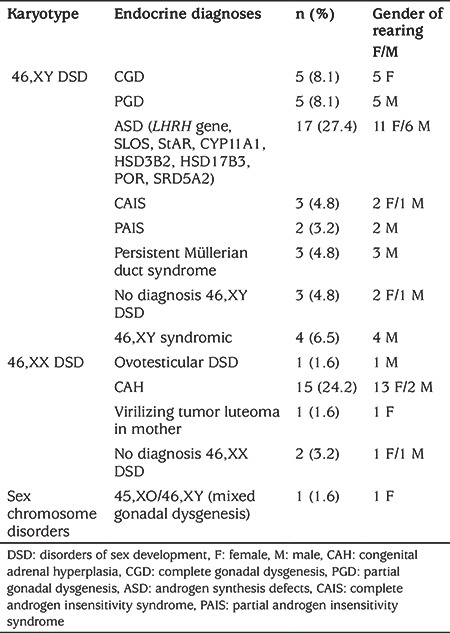
Endocrine diagnoses

**Table 3:**
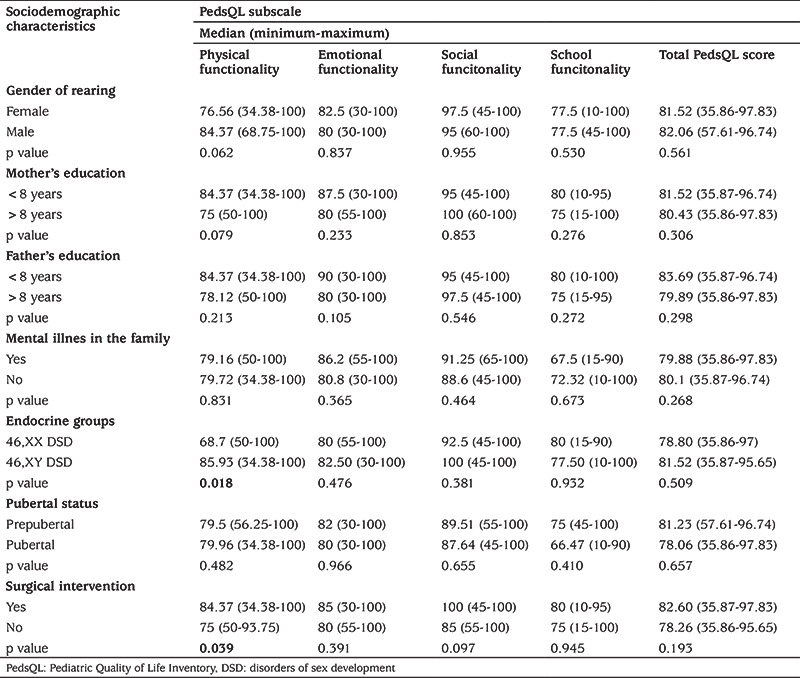
Relationship between children’s Pediatric Quality of Life Inventory scores and sociodemographic characteristics, endocrine groups, pubertal status and surgical intervention

**Table 4:**
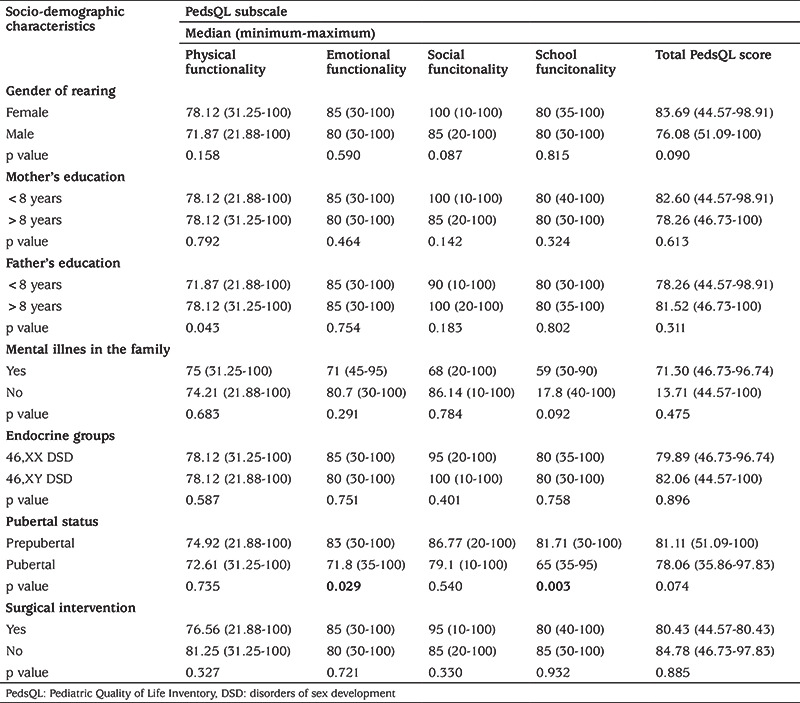
Relationship between parent’s Pediatric Quality of Life Inventory scores and sociodemographic characteristics, endocrine groups, pubertal status and surgical intervention

**Table 5:**
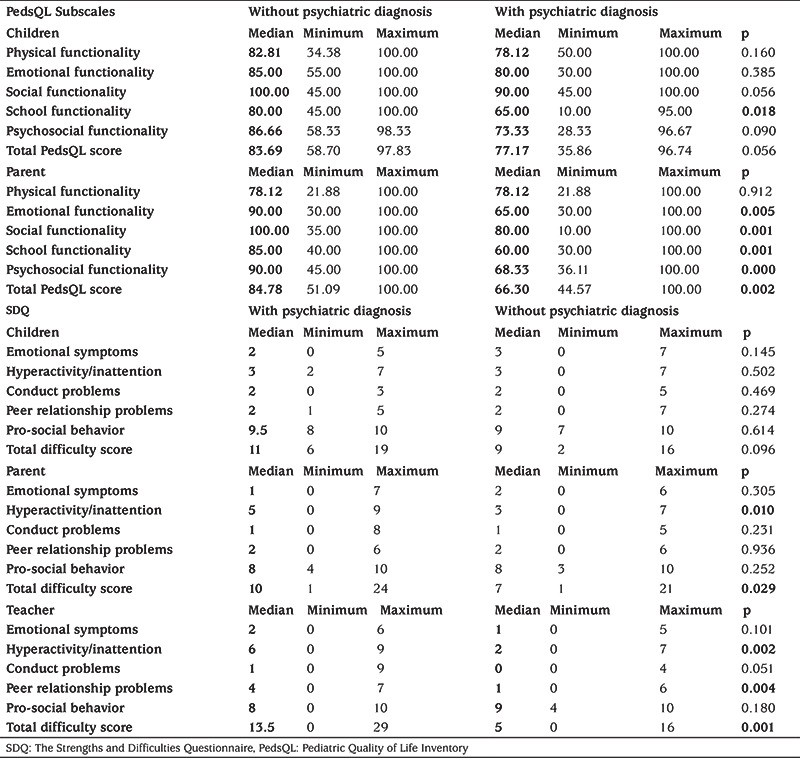
Relationship between psychiatric diagnosis, Pediatric Quality of Life Inventory and the Strengths and Difficulties Questionnaire scores
